# Heritability and recursive influence of host genetics on the rumen microbiota drive body weight variance in male Hu sheep lambs

**DOI:** 10.1186/s40168-023-01642-7

**Published:** 2023-08-29

**Authors:** Weimin Wang, Yukun Zhang, Xiaoxue Zhang, Chong Li, Lvfeng Yuan, Deyin Zhang, Yuan Zhao, Xiaolong Li, Jiangbo Cheng, Changchun Lin, Liming Zhao, Jianghui Wang, Dan Xu, Xiangpeng Yue, Wanhong Li, Xiuxiu Wen, Zhihua Jiang, Xuezhi Ding, Ghasem Hosseini Salekdeh, Fadi Li

**Affiliations:** 1 State Key Laboratory of Herbage Improvement and Grassland Agro-ecosystems; Key Laboratory of Grassland Livestock Industry Innovation, Ministry of Agriculture and Rural Affairs; Engineering Research Center of Grassland Industry, Ministry of Education; College of Pastoral Agriculture Science and Technology, Lanzhou University, Lanzhou, 730020 People’s Republic of China; 2 College of Animal Science and Technology, Gansu Agricultural University, Lanzhou, 730070 China; 3 Lanzhou Veterinary Research Institute, Chinese Academy of Agricultural Sciences (CAAS), Lanzhou, 730046 China; 4 Department of Animal Sciences and Center for Reproductive Biology, Washington State University (WSU), Pullman, WA 99164 USA; 5 Lanzhou Institute of Husbandry and Pharmaceutical Sciences, Chinese Academy of Agricultural Sciences (CAAS), Lanzhou, 730050 China; 6 Department of Molecular Sciences, Macquarie University, Sydney, NSW Australia

**Keywords:** Sheep, Host genetics, Rumen microbiota, Body weight, Heritability, Microbiota GWAS, Microbiability

## Abstract

**Background:**

Heritable rumen microbiota is an important modulator of ruminant growth performance. However, no information exists to date on host genetics-rumen microbiota interactions and their association with phenotype in sheep. To solve this, we curated and analyzed whole-genome resequencing genotypes, 16S rumen-microbiota data, and longitudinal body weight (BW) phenotypes from 1150 sheep.

**Results:**

A variance component model indicated significant heritability of rumen microbial community diversity. Genome-wide association studies (GWAS) using microbial features as traits identified 411 loci-taxon significant associations (*P* < 10^−8^). We found a heritability of 39% for 180-day-old BW, while also the rumen microbiota likely played a significant role, explaining that 20% of the phenotypic variation. Microbiota-wide association studies (MWAS) and GWAS identified four marker genera (Bonferroni corrected *P* < 0.05) and five novel genetic variants (*P* < 10^−8^) that were significantly associated with BW. Integrative analysis identified the mediating role of marker genera in genotype influencing phenotype and unravelled that the same genetic markers have direct and indirect effects on sheep weight.

**Conclusions:**

This study reveals a reciprocal interplay among host genetic variations, the rumen microbiota and the body weight traits of sheep. The information obtained provide insights into the diverse microbiota characteristics of rumen and may help in designing precision microbiota management strategies for controlling and manipulating sheep rumen microbiota to increase productivity.

Video Abstract

**Supplementary Information:**

The online version contains supplementary material available at 10.1186/s40168-023-01642-7.

## Introduction

Sheep weight is the most important growth indicator used in production and is directly related to meat production [1], fat deposition [2], and reproductive performances [3]. Global meat production and consumption continue to grow due to the increased demand driven by population growth, individual economic growth, and urbanization [4]. It is estimated that global meat production will increase 76% by 2050 to meet the increased demand, with half of the global demand for ruminants coming from developing countries, particularly China, the largest producer and importer of sheep meat [4–6]. This thus poses new challenges to increase the sheep meat production. At the same time, the interest of the consumer for low-fat and healthy meat is increasing due to the negative impact of meat fat on human health [7]; however, in sheep, higher weight is often associated with excessive fat deposition [2]. In light of this, to balance yield and healthiness in sheep meat production, there is an urgent need to comprehensively elucidate the mechanisms that control the body weight (*BW*) and to develop new breeding intervention strategies.

Although classical genetics studies suggested heritability of approximately 30 to 60% for sheep BW [8–12], current genetic breeding and selection for BW have been only partially successful because the phenotype represents a complex multifactorial trait. In addition, new research in genome-wide association analysis (*GWAS*) of sheep weight was limited so far due to the small sample sizes, the relative dispersion of SNP chips, and investigations conducted in complex non-laboratory settings such as grazing [13–15]. Therefore, it is necessary to conduct larger sample size GWAS and whole-genome sequencing studies. Furthermore, in addition to breeding and selection, body weight may also be controlled through the regulation of the gut microbiota. In recent years, several studies have supported the role of the commensal microbiota in the gastrointestinal tract in the regulation of the sheep metabolism [16] and immune response [17], and therefore, the hypothesis to influence the sheep BW through the manipulation of the gut microbiota has grown. In this regard, the rumen, which is primarily responsible for digestive and nutrient absorptive functions in sheep, is colonized by a highly complex and anaerobic microbial ecosystem that is able to convert low-nutrient plant material through fermentation into essential and readily metabolites, accounting for up to 70% of host energy requirements [18, 19].

Since the research studies were conducted by *Pomp’s team* [20], there is a growing evidence that in mammals [21, 22], including humans [23–25], and in agriculturally relevant avian hosts [26, 27], the host genetic variation may affect the composition and structure of their gut microbiota. Of particular note, empirical studies on ruminants were focused only on cattle [28–34], while an association between rumen microbiota and the sheep genome has not been demonstrated yet, further leading to a lack of empirical evidence on whether host genetic influences on the rumen microbiota can also affect sheep phenotypes, such as the traits related to BW.

We therefore hypothesized that host genetics may influence the rumen microbial community in sheep, and that the same host single-nucleotide polymorphism (*SNP*) marker could similarly influence sheep weight through a direct mechanism and an indirect one mediated by marker microbiota. To test these hypotheses, we performed whole-genome re-sequencing and rumen microbiota 16S ribosomal RNA (*16S rRNA*) amplicon sequencing from a cohort of 1150 sheep of the same sex, age, and breed, living on the same farm, and raised on the same diet. We characterized the composition of rumen microbiota, and we estimated the heritability. Additionally, the GWAS for rumen microbial features (microbiota GWAS, *mbGWAS*) was conducted to explore the relationship between host genetics and the rumen microbiota in sheep. The host additive genetics and rumen microbial effects were investigated in relation to the sheep BW by estimating heritability (*h*^*2*^) and microbiability (*m*^*2*^, the proportion of the contribution of gut microbiota on host phenotypes), identifying the genetic SNP markers and rumen marker microbiotas significantly associated with BW by GWAS and microbiota-wide association studies (*MWAS*). Finally, the recursive influence of host genomic variations on weight was identified by combining mbGWAS, GWAS, and MWAS results.

## Material and methods

### Animals, phenotypic data and sample collection

**Fig. 1:**
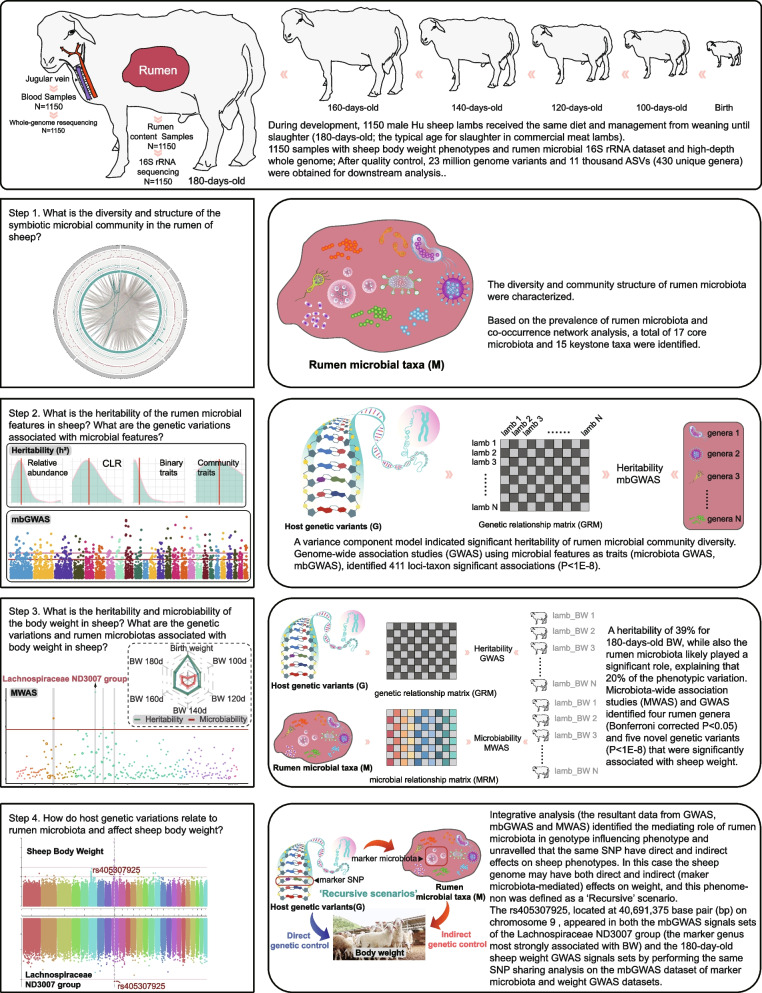
Study design and workflow. This schematic representation highlights, for each step, the research question that we sought to answer, the analysis workflow, the data used, and the generalized result

This study included 1150 male Hu lambs reared on the same farm and divided into four batches over a 24-month period to investigate animal performance and collect the samples (Fig. 1 and Table S1). Specifically, just after weaning, all lambs were transferred to the Minqin experimental farm of Lanzhou University (N38°43′41′′, E103°013′), and they were housed in individual feeding pens (0.8 m × 1.0 m, l **×** w) and fed on the same diet (Supplementary Table 1). For each individual, BW traits were measured with a calibrated livestock scales (at 06:00–08:00 am, before morning feeding) every 20 days, from the age of 100 days to 180 days. At the age of 180 days and after fasting for 12 h, whole blood samples were obtained via the jugular vein and stored at −20 °C until use; each animal was slaughtered according to standard commercial procedures following the requirements of the China Council on Animal Care, and whole rumen content samples were collected immediately and stored at −80 °C. The concentration of VFAs was measured using TRACE-1300 series GC ultra-gas chromatograph (Thermo Scientific, Milan, Italy) by following standard procedures.

### Extraction of host and gut microbial DNA

Host genomic DNA was extracted from blood sample using the whole blood genomic DNA rapid extraction kit (EasyPure Blood Genomic DNA Kit; TIANGEN Bio Company, Beijing, China). All rumen content samples were separately thawed and homogenized again on ice, and then total microbial DNA was extracted from ~200 mg of each sample using EasyPure Stool Genomic DNA Kit (TransGen Biotech, EE301-01, Beijing, China) according to the manufacturers’ instructions. The quality of DNA was assessed on 1% agarose gel electrophoresis. All samples were included in the study, resulting in a total of 1150 host DNA and 1150 rumen microbial DNA samples.

### 16S rRNA gene sequencing and analysis

The V3–V4 regions of bacterial 16S rRNA gene were amplified using specific barcoded primers (341F: CCTAYGGGRBGCASCAG and 806R: GGACTACNNGGGTATCTAAT). Amplicons were sequenced NovaSeq PE250 platform of Illumina (Novogene Biotech Co., Ltd, Beijing, China). Raw sequences were assigned to samples based on their unique barcodes and then trimmed to remove the barcode and primer sequences. The pair-end reads of each sample were assembled using FLASH [35]. The clean sequences underwent quality control analysis using FastQC software (FastQC,http://www.bioinformatics.babraham.ac.uk/projects/fastqc/) and chimeric sequence removal using UCHIME (http://drive5.com/uchime). The filtered data were further processed via DADA2 method [36] in QIIME2 (https://qiime2.org) to produce tables of amplicon sequence variants (ASVs), while the taxonomic assignment was performed using QIIME2 classify-sklearn algorithm by a pre-trained Naive Bayes classifier on 16S Silva database (version 138). To avoid the interference of contingent opportunistic factors and low abundance feature sequence/ASV, the table were filtered using the QIIME2 feature-table filter-features commands (--p-min-samples 3 and --p-min-frequency 5), ensuring that each feature sequence was present in at least 3 samples, and that the total sequencing depth for each feature sequence was greater than 5 reads. Prior to further downstream processing, the data were reduced to the minimum library size (QIIME2 feature-table rarefy commands: -p-sampling-depth 34,736) to obtain the final ASV count data for six taxonomic levels (from phylum to species). Finally, we obtained a preliminary ASV tables of rumen microbes containing 1150 individuals and 11,976 ASVs. These data generated 813 genera. The count data were further normalized to relative abundance by the total-sum scaling method. To further minimize the effect of background noise on downstream analysis, we removed all unclassified genera and then conducted quality control on genera based on the following criteria: (1) mean relative abundance > 0.0001% and (2) occurrence in more than 3 samples. The final genus-level dataset comprises 430 unique bacterial genera.

Alpha diversity metrics, including the Richness, Chao1, ACE, and Shannon index, were calculated based on the ASV table using the “diversity” function in the *Vegan* R package (https://rdrr.io/cran/vegan/). The principal coordinates analysis (PCoA) utilizing the Bray-Curtis dissimilarity matrix was calculated using the “pcoa’” function in the *ape* R package (https://cran.r-project.org/web/packages/ape/index.html).

### Whole-genome sequencing and data processing

The 1150 host qualified DNA samples were subjected to whole genome resequencing at the Novogene facility (Novogene Co., Ltd., China). Each DNA sample was randomly fragmented into 350-bp fragments using a Covaris crusher, followed by library preparation and repairing the ends of the DNA fragments, adding of polyA tails and sequencing adaptors, and PCR amplification according to the manufacturer’s instructions for the TruSeq Nano DNA HT Sample preparation kit (Illumina USA). The PCR amplification products were then purified using the AMPure XP system, initially quantified using Qubit3.0, and the library was diluted to 1 ng/μl. The insert size and effective concentration of the library were then measured using the Agilent 2100 Bioanalyzer and real-time fluorescence PCR, respectively. The selected libraries were sequenced using the Illumina HiSeq X Ten platform (PE150). After resequencing, low-quality reads were removed to obtain high-quality clean data using Trimmomatic (v0.36). We applied the following filtering criteria to eliminate adapters and low-quality bases: reads containing more than 10% unknown nucleotides (N), reads containing more than 50% low-quality bases (*Q*-value < 5), and reads containing more than 10 nucleotides aligned to the adaptor sequence with up to two mismatches. The clean reads were mapped against the sheep reference genome (*Oar_v1.0*) using Burrows-Wheeler-Alignment Tool (BWA) [37] with the command bwa mem -M. Duplicate reads were then marked and removed using SAMBAMBA (https://github.com/lomereiter/sambamba) and indexed in SAMtools (http://github.com/samtools/samtools). Variant detection was performed using the Genome Analysis Toolkit (GATK, https://software.broadinstitute.org/gatk/). Specifically, first gVCF files for each sample using HAplotypeCaller were generated, and then genotypes were called using GenotypeGVCFs. Finally, the SNPs identified were subjected to rigorous quality control using the VariantFiltration module using the following criteria: (1) *QD* > 10.0, (2) *MQ* > 40.0, (3) *FS* < 60.0, (4) MQRankSum > −12.5, (5) ReadPosRankSum > −8.0. Subsequently, the SNP datasets generated above (71,403,155 unfiltered SNP loci) were controlled for quality using vcftools with the following parameters: --remove-indels, --minDP 3, --min-alleles 2, --max-alleles 2, --max-missing 0.3, and --maf 0.05. Following these steps, a total of 23,409,311 SNPs distributed over 27 chromosomes and 1150 sheep were obtained for subsequent analysis (n__autosomal SNPs_ = 23,112,008,Table S10).

### Core and keystone microbiota

The rumen microbial genera with a prevalence [prevalence = (the number of sheep samples in which a specific genus was detected/total number of sheep samples) × 100%] equal to 100% were defined as the core microbiota of sheep, which are the specific rumen genus present in all individuals (*n* = 1150) when they are considered core microbiota. To unravel the patterns of interaction between rumen microbiotas at the genus level, a microbial co-occurrence network was constructed to identify keystone taxa by calculating for each node the within-module connectivity (Zi) and among-module connectivity (Pi) [38, 39]. In details, we excluded rumen genera with a summed relative abundance of less than 0.01% and a prevalence of less than 1.5%, and then we calculated the Spearman correlation factors between microbial genera based on centered log ratio (CLR)-transformed data using the R package *Hmisc* (https://CRAN.R-project.org/package=Hmisc). Correlation coefficients (in absolute value) greater than 0.6 and correlation coefficient matrices with P_adj (Benjamini-Hochberg) less than 0.05 were retained for network building, and networks were constructed in the *igraph* package (https://github.com/igraph/rigraph) followed by visualization as co-occurrence networks in *Gephi* software. Subsequently, we calculated the network modularity and module division using the *igraph* R-package, and we identified the network nodes according to their within-module connectivity (*Zi*) and among-module connectivity (*Pi*). We defined each node as one of four types: (1) peripheral nodes (*Zi* < 2.5, *Pi* < 0.62), (2) connectors (*Zi* < 2.5, *Pi* > 0.62), (3) module hubs (*Zi* > 2.5, *Pi* < 0.62), and (4) network hubs (*Zi* > 2.5, *Pi* > 0.62). Connectors, module hubs, and network hubs are generally considered to be the keystone genera.

### Investigation of the association between host genetics and the rumen microbiota

#### Mantel test

Based on the filtered SNPs dataset, we used the method proposed by Yang et al. [40] to construct a genetic relationship matrix (*GRM*) of 1150 individuals using GCTA v1.94.1 software [41]. Later, a microbial relationship matrix (*MRM*) of these 1150 individuals was constructed based on a normalized (*z*-value) dataset of rumen microbial ASVs using the formula described by Wen et al. [42] and a customized R script by Tang et al. [43]. Based on this, we used the Mantel test to search for the correlation between GRM and MRM using Pearson correlation (9999 permutations).

#### Estimation of rumen microbial heritability

Subsequently, we conducted an estimation of rumen microbial heritability (h^2^) at the genus level. We first excluded rumen genera that were present in less than 1.5% of the sheep samples, resulting in the retention of 290 genera. Subsequently, we classified microbial traits using a threshold of 60% prevalence, as described by Wen et al. [26]. Genera present in less than 60% of the sheep samples were considered binary genera traits (present or absent), while genera present in more than 60% of the samples were considered quantitative genera traits (relative abundance). In addition, we produced a CLR transformation for quantitative traits data to avoid spurious results. We estimated h^2^ of 300 microbial features (5 alpha indexes, top 5 PCoA scores, 209 binary traits and 81 quantitative traits) based on GRM using restricted maximum likelihood (REML) analysis implemented in GCTA and using birthplace, rearing season, and the top five principal components (PCs) as covariates. The estimation model is as follows:

1 $${\varvec{y}}=\mathbf{X}\mathbf{b}+\mathbf{W}\mathbf{a}+\mathbf{e}$$

In the above equation, $y$ is the vector of observations for rumen microbial traits;$b$ is the vector of fixed effects;$a$ is the vector of additive genetic effects following a distribution of $\mathbf{N}(0,\mathbf{G}{\upsigma }_{{\varvec{a}}}^{2})$**,** where $G$ is GRM and ${\sigma }_{a}^{2}$ is the additive genetic variance; and $e$ is the vector of residual effects following a distribution of $\mathbf{N}\left(0,\mathbf{I}{\upsigma }_{e}^{2}\right)$, where $\mathbf{I}$ is an identity matrix and ${\sigma }_{e}^{2}$ is the residual variance. $X$ and $W$ are incidence matrices for $b$ and $a$, respectively. The h^2^ was estimated as $\frac{{{\varvec{\sigma}}}_{{\varvec{a}}}^{2}}{{{\varvec{\sigma}}}_{{\varvec{p}}}^{2}}$, where ${\upsigma }_{p}^{2}$ is the phenotypic variance. A likelihood ratio test (*LRT*) was used to test whether the heritability of a given phenotype was significant (*P*_LRT_ < 0.05).

#### Microbiota genome-wide association studies (mbGWAS)

For significantly heritable microbial features above (h^2^, *P*_LRT_ < 0.05), a mbGWAS analysis was performed using the mixed linear model (*MLM*) implemented in the *rMVP* R package [44] (https://github.com/xiaolei-lab/rMVP). To minimize potential sources of bias in the analyses, we excluded SNPs located on the sex chromosomes for mbGWAS. Furthermore, to avoid the risk of false positives, we excluded bacterial genera with a prevalence below 30% before conducting mbGWAS for binary traits. Finally, we carried out mbGWAS to detect SNP-microbial features associations for 80 heritable microbial features (h^2^, *P*_LRT_ < 0.05,nine heritable microbial community traits, 64 heritable quantitative genera traits, and seven heritable binary genera with a prevalence ranging between 30 and 60%) using 23,112,008 autosomal SNPs and 1150 animals. For each mbGWAS, the model included birthplace, rearing season, and top three PCs as covariates. We first used a genome-wide suggestive significant *P*-value threshold of 1 × 10^−6^ to select marker genetic variants that showed association with microbial features. This threshold was selected to maximize the strength of genetic instruments. Subsequently, we set a genome-wide significant threshold of 1 × 10^−8^, along with a Bonferroni-adjusted study-wide significance level of 0.05/N_SNPs_ (0.05/23,112,008 = 2.163378e-09) for significant associations. We used Ensemble Variant Effect Predictor (VEP; https://www.ensembl.org/vep) for variant annotation and functional annotation.

### Evaluating effects of host genetics and the rumen microbiota on body weight

#### Heritability and microbiability

To gain a better understanding of the relative contribution of host genetics and rumen flora to the sheep BW variation, the h^2^ and microbiability (*m*^*2*^) were examined for all weight phenotypes using the GRM and MRM described above, respectively. Phenotypic heritability was first estimated using a same model to that used for the rumen microbial heritability, as described above. However, in this case, the trait of interest ($y$) in the model [31] represents the vector of observations for weight phenotypes. The m^2^ was the concept that corresponds to the heritability, and it was used to assess the proportion of the total phenotypic variance explained by the gut microbiota [45, 46]. The estimation model is as follows:

2 $${\varvec{y}}=\mathbf{X}\mathbf{b}+\mathbf{Z}\mathbf{m}+\mathbf{e}$$

In the above equation, $y$ is the vector of observations for weight traits; $m$ represents the random effect of rumen microbiota following a multinomial distribution of $\mathbf{N}(0,\mathbf{M}{\sigma }_{m}^{2})$, where $M$ is the MRM and ${\sigma }_{m}^{2}$ is the rumen microbial variance; $Z$ is incidence matric for $m$. The m^2^ was estimated as $\frac{{{\varvec{\sigma}}}_{{\varvec{m}}}^{2}}{{{\varvec{\sigma}}}_{{\varvec{p}}}^{2}}$. Other parameters were consistent with the parameters in model 1 described above. In this study, it referred specifically to the part of the weight phenotypic variation caused by rumen microbiota, as operationalized by using MRM instead of GRM in GCTA.

Here, we considered both sources, host genetics and rumen microbiome, as simultaneous components of the total phenotypic variation. Briefly, we expanded the model by incorporating both the GRM and MRM as input matrices. The extended estimation model is represented as follows:

3 $${\varvec{y}}=\mathbf{X}\mathbf{b}+\mathbf{W}\mathbf{a}+ \mathbf{Z}\mathbf{m}+\mathbf{e}$$

The parameters were consistent with the parameters described above.

#### GWAS and microbiota-wide association studies (MWAS)

Subsequently, we pinpointed the marker SNPs and marker microbiotas associated with body weight at 180 days of age in sheep. Phenotypic GWAS were conducted using the method described in mbGWAS, with a significant genome-wide *P*-value threshold of 1 × 10^−8^ and a suggestive significant genome-wide *P*-value of 1 × 10^−6^. As for marker rumen genera, the filtered genus-level dataset described above was used, and statistical analyses on the associations between these 290 genera and body weight at 180 days of age were performed in R using the two-part microbiota-wide association model described by Wen et al. [26] and Fu et al. [47]. The first part of the model in our study targeted binary traits, which were named as binary traits based on the presence or absence of the bacterial genus. Specifically, a relative abundance greater than zero was coded as 1 (present), while equal to zero was coded as 0 (absent), obtaining a sample prevalence of less than 60%. The second part of the model is focused on the quantitative traits, and it is commonly used for regression analyses between phenotype and abundance of bacterial genera with sample prevalence greater than or equal to 60%. The MWAS model is described as follows:

$$\left\{\begin{array}{c}y={\beta }_{1}b+e\\ y={\beta }_{2}q+e\end{array}\right.$$

where $y$ is the 180-day-old weight value after adjustment for birthplace and rearing season. The first three PCs were also used as a covariate in the association analyses to adjust for population substructure. ${\beta }_{1}$ and ${\beta }_{2}$ represent the regression coefficients for the binary and quantitative models, respectively. $b$ is the binary trait, $q$ is the CLR-transformed bacterial genus abundance, and *e* is the residual effect. The final calculated *P*-values were subjected to multiple comparisons using a Bonferroni correction, setting the threshold *P* adj-value to 0.05.

## Results

### Landscape of core and keystone rumen microbiota composition

**Fig. 2:**
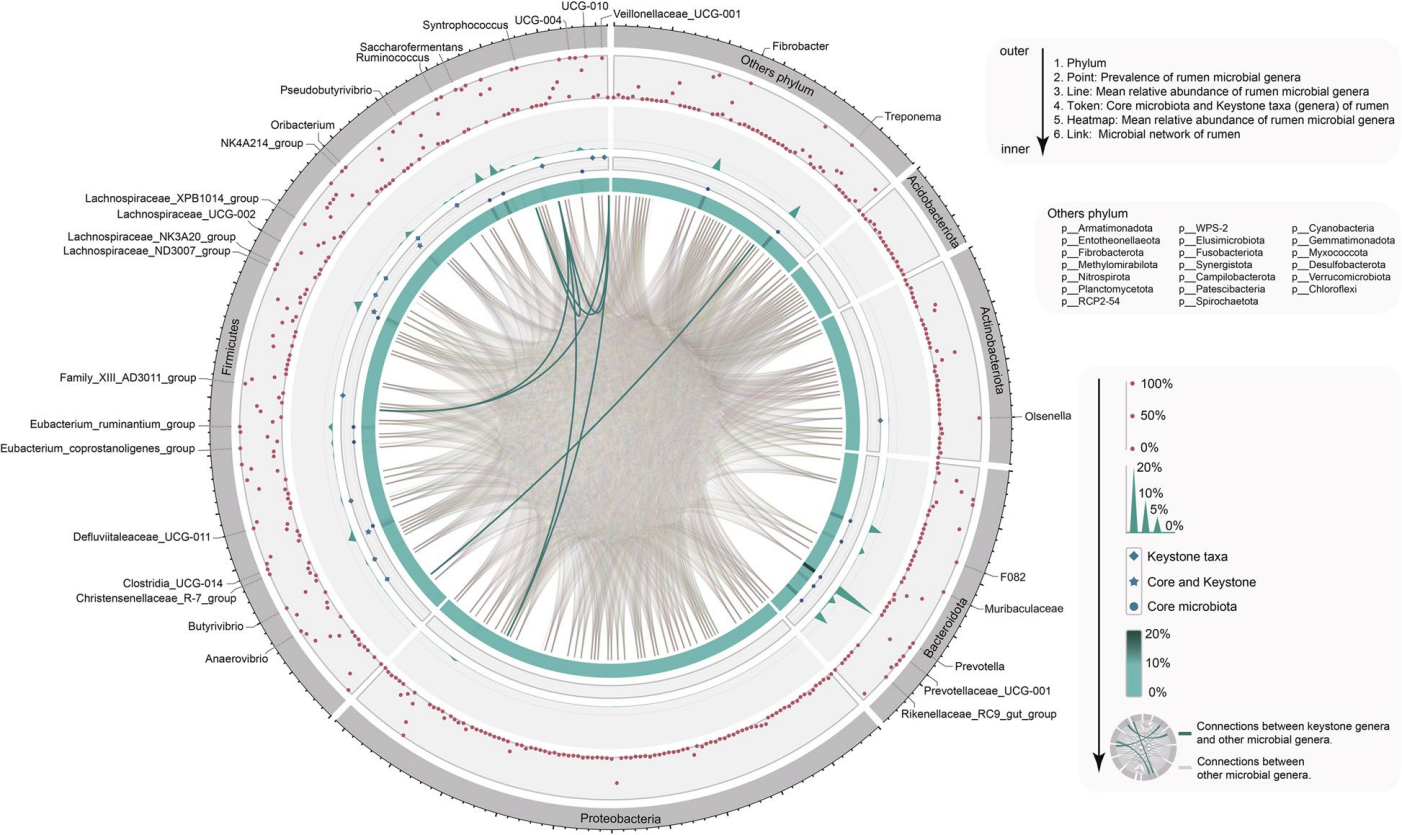
Compositional profile of rumen bacterial communities in sheep at the genus level

To identify symbiotic interactions, if any, between the host and rumen microbiota, the microbial genera that could be critical for the organization and maintenance of the rumen ecosystem were investigated. After low-quality genera were filtered out, a total of 430 unique genera were identified, among which, 17 genera comprised the core rumen microbiota (genera present in 100% of individuals; animal cohort information, see Table S1) and the cumulative abundance accounted for more than 75% of the total microbial abundance in the rumen (including *Christensenellaceae R-7 group*, *Clostridia UCG-014*, *Eubacterium coprostanoligenes* group, *Eubacterium ruminantium* group, *F082*, *Fibrobacter*, *Lachnospiraceae ND3007 group*, *Lachnospiraceae NK3A20 group*, *Muribaculaceae*, *NK4A214 group*, *Prevotella*, *Prevotellaceae* *UCG-001*, *Rikenellaceae RC9 gut group*, *Ruminococcus*, *Saccharofermentans*, *Treponema*, and *Erysipelatoclostridiaceae* *UCG-004*; Fig. 2 and Table S2). A co-occurrence network of rumen microbial communities was analyzed using a Spearman rank correlation coefficient matrix based on significant (*P*_Benjamini-Hochberg_ < 0.05) and strong (|*r*| > 0.60) values to identify co-occurrence patterns. These data were crucial to identify 15 connectors as keystone genera with high connectivity between microbial communities (Fig. 2 and Table S3). Altogether, the genera that were identified showed a pivotal role in the rumen microecosystem as potential drivers of microbiota structure and function, particularly *Christensenellaceae R-7 group*, *Lachnospiraceae NK3A20 group*, and *NK4A214 group*, who were identified in both the core microbiota and keystone taxa.

### Heritability of rumen microbial features

**Fig. 3:**
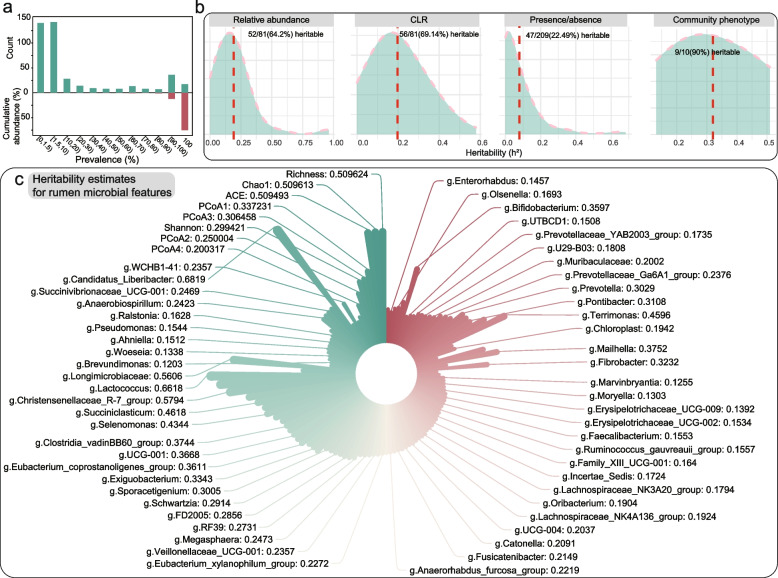
**a** Cumulative relative abundance and number of bacterial genera in the rumen of sheep at different prevalence levels. **b** Percentage of heritable rumen microbial genera in sheep. The red dashed line indicates mean heritability. **c** Heritability estimates for microbial features

To investigate whether host genetics may impact the rumen microbiota, the Mantel test was used to examine the possible associations between the genetic relationship matrix (*GRM*) and microbial relationship matrix (*MRM*). These analyses identified significant associations between the host genome and rumen microbiota (Mantel statistic r: 0.1122, *P*-value: 1e-04), which indicated that host genetics can modulate specific taxa of the rumen microbiota. Subsequently, h^2^ was estimated for 300 rumen microbiota features in 1150 sheep to explore the relative proportion of total variation in microbiota community regulated by host genetics. These included ten measures of microbial community trait [amplicon sequence variant (*ASV*) richness, Chao1, ACE, Shannon index, *Firmicutes:Bacteroidetes* (*F:B*) ratio, and the first five principal coordinates (*PCoAs*) of the Bray-Curtis dissimilarity metric; Table S4]. A total of 81 single-taxon traits (taxa as quantitative traits), representing the relative abundance of individual microbiota genera, were detected at high sample prevalence in over 60% of animals. The h^2^ value was also estimated for 209 binary traits that reflected the presence or absence of a bacterial genus in a sample (limited to genera detected in 1.5–60% of animals, treated as low-abundance rumen microbiota). All microbial genera included in the estimation of h^2^ accounted for over 91% of the total rumen microbial abundance, with single-taxon traits accounting for 89.88% (Fig. 3a).

We found that 64% (52/81) of the quantitative genera and nine community traits were significantly heritable [likelihood ratio test (*LRT*); *P*_LRT_ <0.05)]. The 76% (13/17) of the core genera and 80% (12/15) of keystone taxa were also found among heritable taxa, and a similar proportion was obtained using a centered log-ratio transformation (*CLR*) for the correction of potential constitutive artifacts (69%, the CLR transformed quantitative genera: 56/81). Ultimately, a total of 79% of the high-prevalence genera were heritable [64/81; 82% (14/17) core microbes and 87% (13/15) keystone taxa] (Fig. 3b and Table S5). Binary rumen genera only represented 1.25% of the total abundance but could also be connected to host genome, with 22% (47/209) identified as heritable but were largely limited by their prevalence. These heritable genera showed significant heritability estimates ranging from 0.09 to 0.68 (Fig. 3c); the h^2^ was the highest for *Candidatus Liberibacter* (0.68, *P*_LRT_ = 2.42E-07) which had a very low relative abundance and prevalence, whereas heritability was lowest for *Caldalkalibacillus* (0.09, *P*_LRT_ = 5.00E-02). Among the heritable core genera, *Christensenellaceae* *R-7 group* had the highest heritability (0.58, *P*_LRT_ = 1.07E-08), while *F082* had the lowest value (0.10, *P*_LRT_ = 0.10). Four of these heritable core genera (*F082*, *Muribaculaceae*, *Prevotella*, and *Rikenellaceae RC9 gut group*) belonged to phylum Bacteroidetes, one (*Fibrobacter*) belonged to Fibrobacterota, and the rest were Firmicutes. Possibly the most intriguing observation was that rumen microbial community structure, diversity, richness, and composition overview were all moderate to high heritability traits, with heritability as high as 0.51 for both ASV richness and ASV Chao1 and the first four PCoAs (cumulatively explaining 54.72% of the overall variance in rumen microbiota composition) all having a heritability above 0.2. Members of *Firmicutes* in the rumen represent the most predominant heritable taxa, followed by *Bacteroidota* (Table S5). The cumulative total abundance of heritable genera was as high as 73%, with an average prevalence of 57%. Additionally, the F:B ratio, which can reflect obesity in humans and other mammals, was also heritable (0.15, *P*_LRT_ = 0.02). Overall, these values indicate that rumen microbes are largely heritable, and that the possible effects of host genetics on the rumen core microbiota and keystone taxa appear to be nearly universal.

### Large-scale GWAS identified host genetics that profoundly affect sheep rumen microbiota

**Fig. 4:**
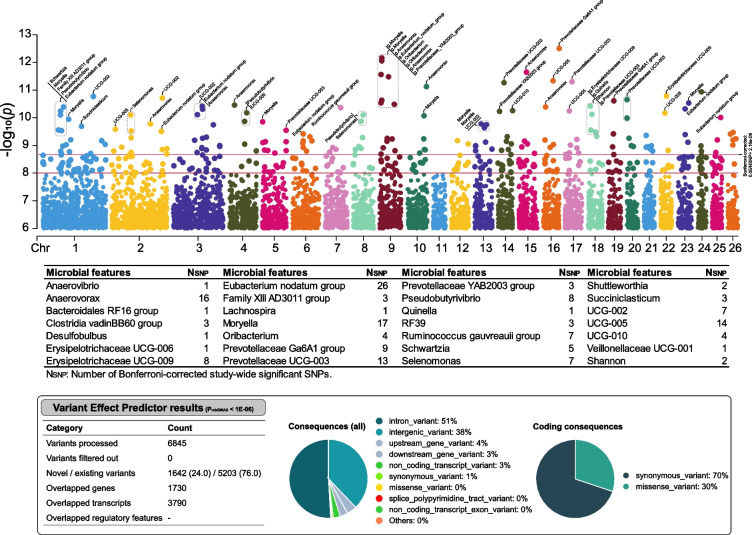
Genome-wide association of sheep genetics and heritable rumen microbial variations. Manhattan plot of host genomic associations with microbial features with at least one genome-wide significant association (*P* < 1 × 10^−6^). The *y*-axis shows the −log10 transformation of the association *P*-value observed at each tested variant. The *x*-axis shows the genomic position of variants. The thresholds of study-wide (0.05/N_SNPs_; *P* = 2.163378e-09) and genome-wide (*P* = 1 × 10^−8^) significance are shown with horizontal lines. The autosome variants were annotated by Ensembl Variant Effect Predictor

To gain a deeper understanding of the impact of host additive genetics on rumen microbiota, we investigated the association between 23,112,008 autosomal genetic variants and 80 rumen microbiota features that were significantly heritable (h^2^, *P*_LRT_ < 0.05) in a cohort of 1150 sheep (see “Material and methods”: “mbGWAS”). Finally, a total of 411 associations involving 405 loci that associated independently with 1 or more of the 80 rumen metabolites at significance (*P* < 1 × 10^−8^). Using a more conservative Bonferroni-corrected study-wide significant *P*-value of 2.163378e-09 (0.05/23,112,008), we were able to identify 171 associations, which involved a total of 171 genomic loci and 28 microbiota features (as shown in Fig. 4 and Table S7).

According to the genome-wide suggestive significant threshold of *P* < 1 × 10^−6^, we identified 6845 SNP loci, of 106 were associated with two or more microbiota features (Fig. 4 and Table S7). Of these, 2709 SNPs were located within the genes. These suggestive significant SNPs were widely and evenly distributed across all autosomal chromosomes (Table S7). The average number of genetic variants for each microbiota trait was 86. The most significant associations were Prevotellaceae Ga6A1 group with the SNP: rs405050318 (*P* = 3.13E-13). Both genetic variants, rs427324596 and rs417318619, exhibit associations with six distinct microbiota traits. For the four alpha-diversity indexes and *F:B* ratio index, a total of 217 SNP loci and 259 associations were observed. Among these, two SNPs (rs427324596 and rs417318619) exhibited significant associations with all four indexes simultaneously. Furthermore, 6423 SNPs were significantly associated with 71 rumen genera (6504 associations), with 51.66% (3318 no redundant loci) of these SNPs associated with *Eubacterium nodatum* group, *Anaerovorax*, *Prevotellaceae UCG-003*, *Moryella*, UCG-005, *Ruminococcus gauvreauii* group, *Prevotellaceae Ga6A1 group*, *Pseudobutyrivibrio*, *RF39*, and *UCG-002*. The selection of candidate genes for heritable rumen microbes further increases the feasibility of inducing a particular microbiota in the production changing the breeding techniques, including the regulation of host phenotypic and production performances based on the control that the heritable rumen taxa may have on animal performances.

### Proportion of variation in sheep body weight explained by host genetics and rumen microbiota

**Fig. 5:**
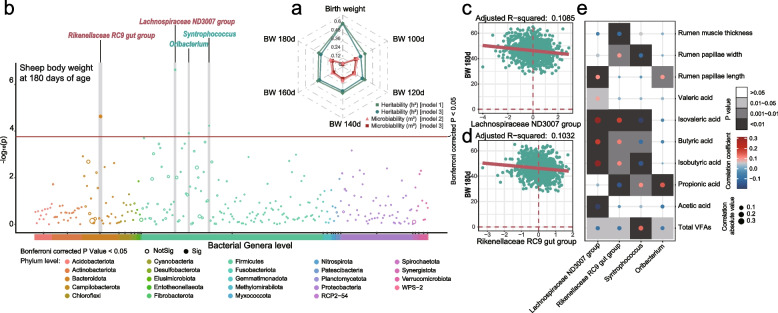
**a** Heritability (h^2^) and microbiability (m^2^) for weight traits in sheep. **b** Manhattan plot shows the results of microbiota-wide association studies (MWAS). Four bacterial taxa were significantly associated with body weight at 180 days of age in sheep (Bonferroni-corrected *P*-value < 0.05). Red labels indicate that the bacterial genus is a core microbe, and green labels indicate that the bacterial genus is a keystone taxon. **c** and **d** A linear model was fitted to identify the association between rumen marker genera and BW. **e** Correlation patterns showing the rumen marker genera associated with rumen volatile fatty acids and rumen morphology

To explore to which extent the host additive genetics and rumen microbiota contributed to weight traits in sheep, we calculated h^2^ and microbiability (*m*^*2*^) estimates for the six BW traits using the same GRM and MRM described above, respectively (Fig. 5a and Table S8). We found all BW traits to have upper moderate to high heritability, ranging from 36 for 100-day-old weight to 61% for birth weight, with a heritability of 39% for 180-day-old weight (Fig. 5a and Table S8). As with heritability, the m^2^ was between 0 and 1 and with increasing m^2^ values, a major contribution of rumen microbiota to the traits was demonstrated. After correction for host genetics, 180-day-old weight had a moderate microbiability estimate of 20%. The m^2^ for birth weight obtained using rumen microbiota data collected at 180-day-old was nonsignificant, and the mean m^2^ for BW at other ages was 20%, ranging from 17% for 100-day-old weight to 24% for 100-day-old weight.

We further considered both host genetics and rumen microbiome as simultaneous components of the total phenotypic variation and calculated the h^2^ and m^2^ of body weight traits. The results from these models were largely consistent (Fig. 5a and Table S8). The combined contribution of host genetics and rumen microbiota to the 180-day-old weight phenotype was 52%. Specifically, the heritability was estimated to be 0.35, indicating the proportion of phenotypic variance attributed to host genetics. Additionally, the microbiota contribution was estimated to be 0.17, indicating the proportion of phenotypic variance attributed to the rumen microbiota.

### Heritable rumen microbes affect sheep weight

Above, we demonstrated that host genetics can influence the rumen microbial community in sheep, and that together with rumen microbiota, may contribute to sheep BW. Later, a two-part model for the MWAS analysis between rumen microbiota and BW in sheep [26, 47] was used to identify which indicator of microbiota taxa may regulate this mechanism. From this analysis, four last genera were identified (Fig. 5b and Table S9), including *Lachnospiraceae ND3007 group*, *Rikenellaceae RC9 gut group*, *Syntrophococcus*, and *Oribacterium*, which were all heritable and significantly associated with 180-day-old weight (Bonferroni corrected *P* < 0.05). Among them, *Lachnospiraceae ND3007 group* and *Rikenellaceae RC9 gut group* emerged as moderately heritable core taxa, explaining 10.85% and 10.32% of variance on 180-day-old weight (Fig. 5c & d), respectively. By contrast, the linear fit correlation coefficients for the potential core and keystone genera *Oribacterium* (prevalence = 99.91%) and *Syntrophococcus* (prevalence = 99.57%) were both over 9.8%.

To investigate the potential relationships among BW-related microbial markers and rumen metabolic profiles, Spearman’s rank correlation analysis was performed between the selected bacterial genera and rumen volatile fatty acids (*VFA*) profile, rumen papillae length and width, and muscle thickness (Fig. 5e). This analysis showed that total VFAs (sum of all individual VFAs) shared a positive correlation with *Syntrophococcus* but were negatively correlated with *Lachnospiraceae ND3007 group*, *Rikenellaceae RC9 gut group*, and *Oribacterium*. Acetic acid was negatively correlated with Lachnospiraceae ND3007 group. Propionic acid levels were positively correlated with *Syntrophococcus* and *Oribacterium* but negatively correlated with* Rikenellaceae RC9 gut group*. Likewise, the abundance of candidate genera was significantly associated with rumen histomorphology. In particular,* Lachnospiraceae ND3007 group* and *Oribacterium* were positively associated with rumen papillae length,* Rikenellaceae RC9 gut group *was correlated with rumen papillae width, *Syntrophococcus* was negatively correlated with rumen papillae width, and *Rikenellaceae RC9 gut group* shared a negative relationship with rumen muscle thickness (Fig. 5e). Overall, these four weight-related indicator taxa and their association with metabolic profiles further emphasized the central role of heritable rumen microbiota, specifically core and keystone taxa, in the host phenotype and rumen microbiota metabolism in sheep.

### Genome-wide association study reveals novel loci controlling sheep weight

**Fig. 6:**
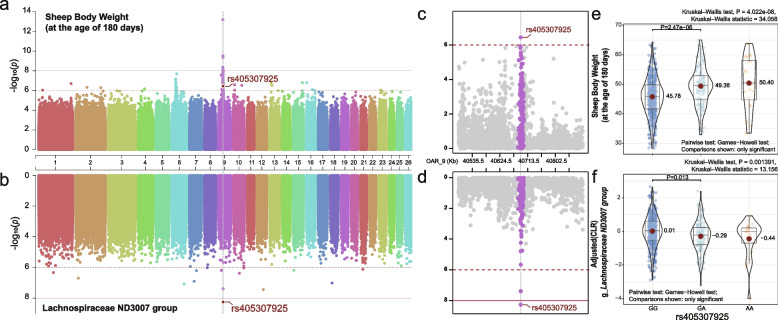
**a** and **c** Genome-wide association analysis (GWAS) for body weight at 180 days of age and *Lachnospiraceae ND3007 group* (the marker genera most strongly associated with 180-day-old weight) in sheep. The horizontal red solid line indicates the genome-wide significance (*P* < 1 × 10^−8^) thresholds. **b** and **d** Magnified view of the region of interest. **e** Comparison of the body weight at 180 days old of different genotypes of the rs405307925 SNP of *TOX*. **f** Comparison of the* Lachnospiraceae ND3007 group* in rumen of different genotypes of the rs405307925 SNP of *TOX*

We next conducted GWAS to identify the SNPs loci in the host genome linked to BW in sheep, using the phenotypic data from the same cohort of 1150 animals and the same autosomal SNPs dataset used for the mbGWAS. A total of five SNP loci were identified to be significantly associated with the body weight of sheep at 180 days old, at a significance level of *P* < 1 × 10^−8^, all located on OAR 9. With a more moderate genome-wide suggestive significant *P*-value of 1 × 10^−6^, we identified 94 SNPs, spanning OARs 1, 2, 4, 6, 9, 10, 13, 15, 16, and 23 (Fig. 6a and Table S10). The most significant association was observed with the intergenic SNP, rs413796993 (*P* = 6.99E-14), on OAR 9 at 38007144bp. The second most significant SNP (rs417240663; *P* = 3E-10) was located on OAR 9 at 38659928bp, in an intron of the *XKR4* gene, which cumulatively harbored 3 significant and 16 suggestive significant SNPs. Notably, we observed that several regions in OAR 6 and OAR 9 together contained more than 84% (79/94) of the marker SNPs. Eight of these loci were located within an ~900-kb region on OAR 6 (33.97–34.88 Mb), including four located in introns of *BMPRIB*, one in introns of *PDLIM5*, and one in introns of *UNC5C*. Additionally, two SNPs were located in a 72.66-kb region between 42.21 and 42.28 Mb on OAR 6, both in the intron of *LCORL*. On OAR 9, a large region spanning ~4.8 Mb (37.42–42.23 Mb) was found to contain 60 SNPs, 33 of which were located within seven genes, including *ATP6V1H*, *LYPLA1*, *RP1*, *XKR4*, *SDCBP*, *TOX,* and *CA8*; a SNP located in *KCNQ3* was also identified on OAR 9. In addition, four of the 15 SNPs on other autosomes were also located in introns, such as rs399594440 in *UNC80* on OAR2, 10-74576409 (variant ID unknown) in *GPC5* on OAR10, rs425587495 in *NRP1* on OAR13, and rs417185097 in *EXT2* on OAR15. Altogether, the analyses described, which were based on a large sample size and with a reduced environmental interference, led to the identification of genetic regions and novel candidate genes that significantly may influence BW in sheep, which provide new molecular markers for breeding efforts to regulate sheep growth and development and thus improve meat production.

### Recursive influence of host genetics on rumen microbiota drive sheep weight

Although host genetics and rumen microbiota jointly contribute to the sheep weight, our results showed that the host genetics and the rumen microbiota are not independent, and that their effects on weight traits are also not independent and unlinked, while there are some weight-associated microbial features that are controlled by the weight-associated genetic variants. In this case, the sheep genome may have both direct and indirect (marker microbiota-mediated) effects on weight trait, and this phenomenon was defined as a “recursive” scenario. To this regard, we investigated the host genetic aspects that may be responsible for this scenario and the SNPs loci that may have both direct and indirect effects on sheep weight. To address this question, the resultant data from GWAS, mbGWAS, and MWAS were combined and analyzed. Based on finding above showing that the four microbial features were significantly associated with 180-day weight (MWAS identified), we found that all had moderate to strong h^2^ (0.16–0.40, *P*_LRT_ < 0.05), and 306 SNPs affecting these four marker genera were identified (mbGWAS identified; Table S11), indicating an “indirect’” scenario for the control of phenotypes by genetic variants, i.e., host genetics indirectly influencing sheep weight through the control of weight-associated microbiota. Subsequently, we compared the overlapping loci of marker microbiota-associated SNPs in “indirect” scenario (mbGWAS identified) and weight-associated SNPs (GWAS identified) to identify host genetics that influence weight through a “recursive” model. Intriguingly, we found that the rs405307925, located at 40,691,375 bp on OAR 9 (Fig. 6, Tables S7–S11), appeared in both the mbGWAS signals sets of the* Lachnospiraceae ND3007 group *(the marker genera most strongly associated with 180-day-old weight) and the 180-day-old weight GWAS signals sets. Therefore, this represents the first time that a “recursive” scenario of genomic markers influencing the phenotypes was identified using a locus overlap algorithm in sheep. To identify more potential “recursive” scenarios, we then used a Kruskal-Wallis test to examine the differences in BW within marker microbiota-associated SNPs (mGWAS identified) genotypes in the “indirect” scenario described above. Finally, we identified six genetic markers (*P*_Kruskal–Wallis test_ < 0.01; Table S12) that have the potential to recursive impact the body weight in sheep. These recursive loci consist of rs405307925, which is situated on the *TOX* gene, as well as rs425633529 and rs604806950, which are located on the *GLRX3* and *PRR14* genes, respectively.

## Discussion

Body weight represents the most economically important trait for sheep production, and it may be strongly influenced by host genetics and rumen microbial ecosystem. However, these studies were only focused on single effects, that is to say only the independent influence of host genome or the microbiota. Therefore, whether the host genetics may interact with the rumen microbiota to influence BW in sheep has remained largely unknown to date. Here, we described a relationship between host genome, rumen microbiota, and BW phenotype in 1150 sheep subjected to the same diet and management conditions. To gain better insights into this topic, we described the core and keystone structure of the rumen microbiota. We calculated the h^2^ of the rumen microbiota, and mbGWAS was performed to identify the influence of host genetics on individual rumen microbial genera. The h^2^ and m^2^ were also estimated for BW, identified host genetics loci, and rumen microbiota affecting BW. The different causal scenarios in which genetic markers may influence the BW phenotype were analyzed using the locus overlap algorithm and Kruskal-Wallis test. To our best knowledge, this is the first study that reports a large association between host genome and rumen microbiota and BW in sheep to date. Our study provided detailed guidelines on the combined use of microbiota and host genome information in order to predict the complex traits and the reliability of parameter inferences.

Mantel tests are a widely used method in microbiome analysis to examine the macroscopic correlations between host genetics and gut microecological variation. In this study, a significant correlation was observed between the host genome kinship matrix and the rumen microbiota relationship matrix, which is consistent with previous findings in human feces [48] and mice [49]. However, it is important to note that the Mantel test used to investigate the impact of host genetics on shaping the overall structure of the rumen microbiome has its limitations. The Mantel test is a matrix-based method that can only detect linear relationships within the matrix while disregarding nonlinear interactions [50]. Furthermore, the rumen microbiota community is highly complex and diverse, with intricate interactions between different species that can influence the magnitude of the correlation coefficient. Therefore, despite the weak but significant correlation observed, it still suggests that the host genetic background plays a role in shaping the structure of the rumen microbiota.

In this study for the first time, the heritability of the rumen microbiota has been investigated on sheep, and it was demonstrated that the rumen microbial community diversity was heritable, consistently with previously studies in cattle [29], demonstrating a higher h^2^ than those reported for cattle. This finding may be attributed to three main factors: firstly, differences between host species,secondly, the more control for environmental factors and covariates in the design and analysis of the current study [21, 51] and thirdly, higher detection of variants than SNP chips following the resequencing. All together*,* these observations also indicated that most of the variation in rumen microbial communities is due to host genetics in sheep. On the other hand, estimating bacterial heritability provided new perspectives in identifying taxa that were closely related to host but previously unidentified, such as *Candidatus Liberibacter* and Longimicrobiaceae, both of which showed a heritability above 50% but for which no host interactions were detected. However, we found that the heritability of the very low-abundance rumen taxa in sheep was limited by their prevalence in the population (most taxa were present in < 10% of samples). This may be due to which the effective sample size and efficacy of genetic analyses, and therefore very large sample sizes and more sensitive microbial community sequencing technologies (e.g., metagenomic and metatranscriptomic approaches), will be beneficial to address this issue in the future.

We next focused on the BW value, which was measured at 180 days of age, the typical age for slaughter in commercial meat lambs. Host genetics explained 39% of the total phenotypic variation, and indeed, the effect of the rumen microbial communities on sheep BW was 20% following the consideration of host genetics. These results highlighted the need for additional comprehensive analyses based on the genome and rumen microbiota. We therefore used MWAS to screen rumen microbes associated with BW, and we identified four heritable bacterial genera. Interestingly, these taxa derived from a core or keystone microbial subsets, suggesting that ecologically important core and keystone taxa play a role in the development of BW phenotypes in sheep. The increase in major VFAs is beneficial for the animal, and *Syntrophococcus* produced major VFAs that are absorbed through the rumen wall to sustain the energy requirements [52, 53]. *Oribacterium* has been found to be an obesogenic bacterium in human and rat studies [54, 55], and the increasing of *Oribacterium* in the rumen of cattle may promote the synthesis of a variety of fatty acids [56]. For example, the changes in propionate concentrations are correlated with the changes in the abundance of most marker genera. Propionate is the main precursor of hepatic gluconeogenesis in ruminants and is effectively involved in their energy balance [57].

By applying large-scale GWAS, two signal peaks on chromosomes 6 and 9 in sheep were observed. The OAR6 signalling peak region included an orthologous region that has been clearly associated with body size in a variety of mammals [58]. We replicated the relationship between the *LCORL* gene and BW in sheep found by *Al-Mamun and colleagues* [59]. Biologically relevant genes localized to the OAR9 signalling peak region were *KCNQ3*, *ATP6V1H*, *LYPLA1*, and *XKR4*. *KCNQ3* modulates M-currents in NPY/AgRP neurons, affecting neuronal excitability and stimulating the physiological appetite, thereby it contributes to the energy balance [60]. The *ATP6V1H* gene is involved in mediating the regulatory bone formation [61], and SNPs in *ATP6V1H* were previously reported to affect feed efficiency in beef cattle [62]. *LYPLA1* has been identified as a potential candidate gene associated with lipid-related biological processes [63]. *XKR4* encodes an XK-related protein in the XK-Kyle blood group complex. *XKR4* variants which were associated with economically important traits such as growth, and feed efficiency, have been widely detected in cattle, but not in sheep [64]. In addition, some interesting regulatory candidates were identified, with *BMPR1B* (also known as *FECB* and *ALK-6*), *TOX*, and *SDCBP* the ones of major interest. Several studies have demonstrated the key role of *TOX* and *SDCBP* on carcass weight, fertility, and feed efficiency traits in cattle [65]. An important gene involved in glucose metabolism is *CA8* [66], while *NRP1* is involved in the regulation of organ development and function [67]. Furthermore, *PDLIM5* encodes a cytoskeleton-associated protein that plays an important role in cell proliferation and differentiation in a variety of tissues and cell types [68], while *PTTG1IP* was previously reported to be a selectively spliced gene associated with chicken muscle development [69]. The *EXT2* gene encodes an essential component of the glycosyltransferase complex required for the biosynthesis of acetyl heparin, which in turn regulates the signalling involved in bone formation [70]. *UNC80* is one of the subunits of the *NALCN* complex, which is associated with global dysplasia [71]. Although our analysis has identified several SNPs and genes previously not reported using the GWAS analysis, functional validation of these variants is required.

Following an integrated analysis of GWAS, mbGWAS, and MWAS results, our study described a recursive scenario of host genetics influencing phenotype, based on the hypothesis that the same SNPs are directly associated with animal phenotype able to influence the composition of the rumen flora and consequently also the host phenotype. We identified a recursive scheme in which rs405307925, located within the *TOX* gene, affects sheep BW, and its indirect effects are mediated through the *Lactococcus* *ND3007 group*. Notably, this is also the first report in which the SNPs discovered in the target phenotypic GWAS were overlapped with those in the GWAS of microbes associated with the target phenotype. Here, although the biological function of the *TOX* gene in sheep has not yet been reported, it is noteworthy that three previous studies described the key role of the *TOX* gene in regulating the immune T -cell function [72–74]. Although rumen microbes play an important role in host immunity and metabolism, multiple immunological mechanisms are put in place by the host to avoid microbial ecological dysregulation. Therefore, we hypothesized that the host genetics may regulate the immune system, controlling the complex rumen microbes. Future research will be needed to elucidate these complex mechanisms.

This study has some limitations. While it was conducted using the most dominant breed of sheep in China, Hu sheep, generalizing the findings to other breeds, may require additional considerations. As all study animals were males, the applicability to other genders of sheep may be limited. Additionally, the exact mechanism underlying the “recursive” scheme remains elusive. In future studies, a combination of metabolomics, metagenomics, and gene editing techniques will be employed to investigate the mechanisms involved in this recursive scenario.

## Conclusions

In this study, we explored the association of host genetic variation and rumen microbiota and its impact on sheep BW. Using large-scale GWAS and MWAS, novel candidate genes and heritable rumen indicator taxa that potentially affect sheep BW were identified. Furthermore, our results supported the hypothesis that host genetics influences the composition of the rumen microbiota, with an intricate network involving host genetics, rumen microbiota, and sheep weight traits. Additionally, we identified six SNP loci that influence sheep BW through a “recursive” model. Of particular interest is rs405307925, located within the *TOX* gene, which can affect BW both directly and indirectly by influencing the relative abundance of the* Lachnospiraceae ND3007 group*, which in turn affect the BW through the production of VFAs. Our work is the first study that explored the host genetic-rumen microbiota-phenotype relationships on a large-scale sheep population, which was sufficiently attenuated from other factors such as environment and diet. These observations will provide a reliable and detailed guide that can be helpful for the combined use of rumen microbiota, and genomic information useful for predicting complex traits and parameter inferences, which may be useful for developing breeding strategies to improve sheep BW.

### Supplementary Information

**Additional file 1:** **Table S1.** Diet and Animal cohort information. **Table S2.** Prevalence (detection rates) and mean relative abundance of rumen microbiota genera in a single large-scale homogeneous population-based cohort of 1,150 sheep. **Table S3.** Keystone taxa (rumen microbiota genera) in a single large-scale homogeneous population-based cohort of 1150 sheep. **Table S4.** The microbial alpha diversity indices and beta diversity. **Table S5.** Heritability of rumen microbiota. **Table S6. **Single nucleotide polymorphisms (SNPs) information. **Table S7.** Genome-wide association of sheep genetic and rumen microbial variations. **Table S8.** Heritability and Microbiability of Body Weight Traits in Sheep. **Table S9.** microbiome-wide association study (MWAS). **Table S10.** GWAS for sheep body weight at 180 days old. **Table S11.** Microbiota mediated indirect scenarios. **Table S12.** 'recursive' scenarios.

## Data Availability

Raw data were deposited in the National Center for Biotechnology Information database under BioProjectID PRJNA867677. Further data requests and informations can be obtained  by contacting the corresponding author Weimin Wang at wangweimin@lzu.edu.cn.
